# Activity Diversity and Well-Being in Daily Life: Evidence for Heterogeneity Between Older Adults

**DOI:** 10.1093/geronb/gbae025

**Published:** 2024-03-30

**Authors:** Minxia Luo, Robert Glenn Moulder, Laura K Breitfelder, Christina Röcke

**Affiliations:** University Research Priority Program “Dynamics of Healthy Aging,” University of Zurich, Zurich, Switzerland; Department of Psychology, University of Zurich, Zurich, Switzerland; Institute of Cognitive Science, University of Colorado Boulder, Boulder, Colorado, USA; University Research Priority Program “Dynamics of Healthy Aging,” University of Zurich, Zurich, Switzerland; University Research Priority Program “Dynamics of Healthy Aging,” University of Zurich, Zurich, Switzerland; Healthy Longevity Center, University of Zurich, Zurich, Switzerland; (Psychological Sciences Section)

**Keywords:** Ambulatory assessment, Health, Healthy aging, Positive and negative affect, Time use

## Abstract

**Objectives:**

Although higher activity diversity is associated with higher well-being at the between-person level, it is unknown whether a day with higher activity diversity is related to higher well-being within persons. Within 24 hr per day, there are a limited number of activities on which individuals could spend their time and energy. Personal resources could influence the expenditure of energy and thus the experience with daily activities. This study examined daily associations between activity diversity and well-being and whether age and self-related health moderated the associations.

**Methods:**

For seven times per day over 2 weeks, 129 retired older adults (*M*_age_ = 73.9 years, *SD*_age_ = 5.6) reported their present activity engagement and positive and negative affect. Daily activity diversity was operationalized as the number of different activity types reported per day. Daily positive and negative affect were assessed as the average of a range of high- and low-arousal affective states. Self-rated health was assessed with an item from the 12-Item Short-Form Health Survey at baseline.

**Results:**

Multilevel models showed that daily activity diversity was unrelated, on average, to daily positive or negative affect at the between- and within-person levels. Daily activity diversity was associated with lower daily positive affect in participants with lower self-rated health, but the Johnson-Neyman regions of significance were outside of the range of observed data.

**Discussion:**

Divergent patterns were observed in the within-person associations between activity diversity and well-being across participants. Results are discussed in the context of time use and well-being in older age.

One of the continuous and important pursuits in gerontological research is to understand the role of activity engagement in daily life for healthy and successful aging (Horgas et al., 1998; Möwisch et al., 2022). Decades ago, the activity theory proposed that staying active and engaged within life and society is the key to older adults’ well-being (Havighurst, 1963). *Activity diversity* denotes the variety of activity types that people engage in during a particular time frame. It has been shown that activity diversity is associated with higher cognitive functioning (Brown et al., 2023), more agreeable personality states (Lindner et al., 2023), and lower likelihood to develop frailty (Takahashi et al., 2023) and depression (Lee, Yu, et al., 2018).

Does a lifestyle characterized by diversity also foster well-being, one of the key indicators of healthy aging (World Health Organization, 2015)? One study examined two-wave 10-year longitudinal population survey data and showed that, cross-sectionally, older adults with higher activity diversity indeed had higher eudaimonic well-being (e.g., purpose in life); and that, longitudinally, an increase in activity diversity was marginally associated with an increase in positive affect (Lee, Koffer, et al., 2018). Relatedly, higher perception of variety experienced in life is shown to be associated with higher quality of life of older adults at the between-person level (Jansen et al., 2023). Further, an experience sampling study showed that higher activity diversity was associated with more positive mood at a 3-hr time window within older adults (Fingerman et al., 2020). Thus, engagement in diverse activities might promote older adults’ well-being. Prior studies on activity diversity proposed that activity diversity may offer opportunities for social integration and thus benefit well-being (Lee, Koffer, et al., 2018; Lee, Urban-Wojcik, et al., 2022). Moreover, diverse activities might compensate for limited benefits of single activities for well-being (Joseph Sirgy & Wu, 2009; Luo, Macdonald, et al., 2022).

However, it remains unclear whether these associations would also hold for the within-person day-to-day level. Within 24 hr per day, there are a limited number of activities that individuals can spend their time and energy on. The selective optimization with compensation model proposes that older adults would select activities that are of high priority to their life goals and augment resources for these activities (Baltes & Carstensen, 1996). Similarly, the socioemotional selectivity theory predicts that emotional regulation through familiar social contacts becomes increasingly more important in older age—particularly for those with a limited future time perspective—and, thus, the desire of older adults to affiliate with familiar people increases (Carstensen, 1995). These theoretical propositions view older adults as self-regulatory agents who make their own decisions on daily activities and suggest that older adults are selective in choices on which they spend their time and energy. In turn, activity diversity, which might be at odds with an increase in being selective in overall and particularly social goals and activities, may not have a positive association with older adults’ well-being at the within-person level within a day.

Among older adults, those of more advanced age reduce their engagement in physical, social, and cognitive activities more than those of relatively younger age (Buchman et al., 2014; Finkel et al., 2018). Further, older adults with more functional limitations engage in fewer leisure activities (Janke et al., 2006) and have lower activity diversity (Lee, Ng, et al., 2022). Thus, older age and lower health status may encourage older adults to adapt their behaviors, being more selective in their daily activities and devoting more time on fewer rather than more diverse activities. These older adults, with lower resources and possibly lower expenditure of energy, might experience greater enjoyment and well-being when their days are less diverse.

## The Current Study

This study had two research aims. First, we aimed to examine associations between daily activity diversity and daily well-being. We expected that activity diversity would be positively related to well-being at the between-person level and we explored the daily associations between activity diversity and well-being at the within-person level. Second, we aimed to examine whether age and health status moderated these associations. We expected that participants with older age and lower health status would show negative associations of activity diversity with emotional well-being.

This study conceptualized that activity diversity could give rise to well-being. Nevertheless, we acknowledge that positive and negative affect could also, in reverse, govern the choice of everyday activities (Sardina et al., 2022; Taquet et al., 2016). Activity engagement and affect could also have a bidirectional association (Ruissen et al., 2022). We examined data from a larger interdisciplinary study on mobility, activity, and social interactions of community-dwelling older adults from Switzerland (Röcke et al., 2023). We focus our examination on participants who were fully retired because they could have very different time use and experiences of leisure participation than older adults with work and family obligation (Freund, 2020).

## Method

### Participants

This study included 150 older adults living in Switzerland, recruited through the research institute’s participant database, snowballing method, and advertisements in local newspapers. Inclusion criteria were a minimum age of 65 years, sufficient vision to operate a smartphone, computer and internet access at home, and no cognitive impairment (Mini-Mental State Examination score ≥27). We retained the 129 (86%) participants who were retirees, excluding one participant working full time and 20 participants working part time (see Author Note 1). The sample (*M*_age_ = 73.9 years, *SD* = 5.6, range = 65–91) was exclusively Caucasian, consisted of 49% men and 56% married, and had an average of 13.87 years of education (*SD *= 3.45). On average, participants rated their own health as “very good” (*M* = 2.32 on a scale from 1 [“excellent”] to 5 [“poor”], *SD* = 0.82). In total, 127 (98%) participants were rated mildly or not impaired in the baseline assessment using the Short Physical Performance Battery (Guralnik et al., 1994). Participants received 200 Swiss francs as compensation for their participation in the entire study.

### Procedure

The study was conducted according to the Declaration of Helsinki and approved by the Ethics Committee of the Faculty of Arts and Social Sciences of the University of Zurich (permission no. 17.2.4). Written informed consent was obtained from all individuals. Participants completed baseline questionnaires and 2-week smartphone-based ambulatory assessment. The latter included seven prompts per day to report their current activity and positive and negative effects on a smartphone that was provided for the study. The prompts were scheduled every 120 min with a random interval of plus or minus 0–15 min (i.e., each day around 8:30, 10:35, 12:40, 14:45, 16:50, 18:55, and 21:00). All prompts last around 5 min, with the last prompt in the evening lasting around 7 min as it included a longer questionnaire on the experience of the whole day. We examined a total of 1,994 days’ data (97%) out of 2,064 (129 participants × 16 days) possible days.

### Measures

#### Activity diversity

Participants answered the question “[w]hat is your current activity?” by choosing one of 13 different types of activities (see Table 1). The activity types were selected based on prior studies of daily experiences (Fisher et al., 2012; Hultsch et al., 1999). Three members of the study team collected a longer list of possible activities and then collapsed and shortened this list to provide relevant activities in the target group and a list that seemed feasible for smartphone assessment without too much scrolling and the intended target group of older adults. Daily activity diversity was operationalized as the number of different activity types across all assessment points in a day (range = 1–7). Based on our previous research using the same data set (Luo et al., 2023), the count measure and Shannon’s (1948) entropy measure, which promise to take into account the evenness of frequency of each activity (Lee, Koffer, et al., 2018), were highly correlated (*r* = 0.98). We thus took a simpler approach and operationalized activity diversity by counting the number of different activity types.

**Table 1.** Descriptive Statistics of the Key Time-Varying Daily Variables

Variables	*M*	*SD*	Intraclass correlation coefficients
Activity diversity	3.79	1.22	0.21
Positive affect	4.50	0.79	0.74
Negative affect	0.55	0.60	0.64
Activity frequency			
(1) Housework	0.89	1.03	0.14
(2) Cook/eat	0.84	0.85	0.16
(3) TV/music	0.63	0.76	0.23
(4) Education/intellectual	0.77	0.97	0.23
(5) Cultural/religious	0.06	0.25	0.06
(6) Hobbies	0.13	0.46	0.20
(7) Social	0.51	0.82	0.12
(8) Sports	0.24	0.58	0.19
(9) Walk	0.14	0.40	0.12
(10) Doctor/care	0.11	0.35	0.07
(11) Work/volunteer	0.19	0.59	0.22
(12) Rest	0.38	0.65	0.14
(13) Other	0.53	0.87	0.14

*Notes: SD* = standard deviation. *n* = 1,994. (1) Housework = housework/gardening/administrative tasks (at home)/shopping; (2) cook/eat = cooking/preparing food/eating; (3) TV/music = watching TV/listening to music; (4) education/intellectual = education/intellectual stimulation (e.g., reading, puzzles, and further education); (5) cultural/religious = cultural/religious activity (e.g., museum, cinema, and church); (6) hobbies = hobby (e.g., crafts, handicrafts, and making music); (7) social = social interactions (e.g., conversations and visits); (8) sports = sporting activity; (9) walk = going for a walk; (10) doctor/care = visiting the doctor/personal care (incl. e.g., hairdresser); (11) work/volunteer = work/voluntary work; (12) rest = doing nothing/resting; (13) other = other activity.

#### Well-being

On a 7-point scale (0 = not at all to 6 = very strongly), participants rated their positive affect across five items: content, awake, relaxed, happy, and balanced. Positive affect was the average of the five items (omega_within_ = 0.85, omega_between_ = 0.98). Likewise, participants rated their negative affect over the following eight items: unwell, restless, without energy, nervous, worried, annoyed, sad, and angry (omega_within_ = 0.85, omega_between_ = 0.99), with responses being averaged over these items at each prompt. The items were drawn from the PANAS-X (Grühn et al., 2010; Watson et al., 1988) and the momentary version of the Multidimensional Mood Questionnaire (Wilhelm & Schoebi, 2007) to cover a broad range of positively and negatively valanced states that also differed in arousal. Daily positive affect and daily negative affect were computed as the respective average over the seven positive and negative affect ratings.

#### Age

Age was assessed as years since birth.

#### Self-rated health

Self-rated health was assessed with an item from the 12-Item Short-Form Health Survey: “[i]n general, would you say your health is…” (1 = excellent to 5 = poor; Ware et al., 1996).

#### Covariates

We controlled for covariates that are known to influence daily activity engagement and daily well-being, including sex (1 = men, 0 = women), education (years of education), marital status (1 = married/long term partnership, 0 = single/divorced/widowed), and weekend (1 = yes, 0 = no; Kim & Fingerman, 2022). We controlled for daily activity frequency of different activities to ensure that the effects of activity diversity did not come from potential overlap with high frequency of activity engagement (Okabe-Miyamoto et al., 2023). Daily activity frequency was the number of occurrences of each of the 13 activities reported per day. Table 1 shows the descriptive statistics of daily frequency of each activity. For example, across participants, “sports” was on average endorsed 0.24 times per day (*SD* = 0.58). We controlled for study day since the study began (range = 0–15) for potential time-related trends in well-being over the study period.

### Statistical Analysis

We used multilevel models (Bolger & Laurenceau, 2013) to examine the two-level data, where days are nested within participants. We estimated daily well-being as a function of daily activity diversity, and separated the time-varying variable of daily activity diversity into between- and within-person variations. The between-person variation was the average value of each participant’s daily activity diversity, representing each participant’s typical activity diversity. The within-person variation was the raw daily activity diversity score minus the between-person variation (person-mean centered), representing the deviation of activity diversity of a given day from the person’s typical activity diversity. We controlled for study day.

Next, we added age (grand-mean centered), self-rated health (grand-mean centered), and the covariates, including the binary variables of sex, marital status, and weekend and years of education (grand-mean centered) and daily activity frequency of the 13 different activities (within-person variation). Consequently, the reference category of these models were the days, which were the first study days, weekday, and of participants’ own typical activity diversity and activity frequencies, and participants who were women, unmarried, had a sample average age, self-rated health, year of education, and activity diversity. We estimated the random intercept and random slope of the daily activity diversity (within-person variation). We did not estimate the random slopes of daily activity frequency (within-person variation) because the models with the 13 additional random slopes were too complex to converge.

Analyses were conducted in R Version 4.3.2 (R Core Team, 2013) using the “lme4” Version 1.1-35.1 (Bates et al., 2007) and “lmerTest” package Version 3.1-3 (Kuznetsova et al., 2017). We handled missing data with the default setting of the R “lme4” package: restricted maximum likelihood. Pseudo-R-squared indicating explained variance was calculated with the R “MuMIn” package version 1.47.5 (Barton & Barton, 2015). The Johnson-Neyman technique was used to examine the region of significance of the moderating effects (Preacher et al., 2006), the result of which was plotted with the R “interactions” package version 1.1.5 (Long & Long, 2019). Statistical significance was evaluated at *p* < .05.

## Results

Table 1 shows the descriptive statistics of the time-varying daily variables. The intraclass correlation coefficients show that there was more variance across days *within* individuals in the activity variables, whereas there was more variance *between* individuals in the well-being variables. Bivariate between-person associations of the focal variables can be found in Supplementary Table S1.

As shown in Table 2, within persons, higher daily activity diversity on average was not associated with daily positive affect (Model 1, *b* = 0.01, *SE* = 0.01, *p* = .580), but was associated with lower daily negative affect (Model 3, *b* = −0.02, *SE* = 0.01, *p* = .033). However, the latter significant association became nonsignificant once the covariates were added to the model (Model 4, *b* = 0.002, *SE* = 0.01, *p* = .838). Additionally, activity diversity was not associated with positive and negative affect at the between-person level (Models 1–4).

**Table 2.** Within-Person Associations Between Daily Activity Diversity and Daily Well-Being

Parameters	Model 1 Positive affect		Model 2 Positive affect		Model 3 Negative affect		Model 4 Negative affect	
	*b*	*SE*	*b*	*SE*	*b*	*SE*	*b*	*SE*
Fixed effects								
Intercept	**4.52**	0.06	**4.33**	0.09	**0.66**	0.05	**0.78**	0.07
Diversity WP	0.01	0.01	−0.00	0.01	**−0.02**	0.01	0.00	0.01
Diversity BP	0.14	0.09	0.10	0.09	0.01	0.07	0.04	0.06
(1) Housework			0.01	0.01			−**0.04**	0.01
(2) Cook/eat			0.01	0.01			−**0.04**	0.01
(3) TV/music			0.00	0.02			−**0.03**	0.01
(4) Education/intellectual			0.01	0.01			−**0.05**	0.01
(5) Cultural/religious			0.03	0.04			−0.05	0.04
(6) Hobbies			0.02	0.02			−0.03	0.02
(7) Social			**0.03**	0.01			−**0.03**	0.01
(8) Sports			**0.08**	0.02			−**0.05**	0.02
(9) Walk			**0.07**	0.03			−**0.07**	0.02
(10) Doctor/care			−0.05	0.03			0.02	0.03
(11) Work/volunteer			−0.02	0.02			−**0.04**	0.02
(12) Rest			−**0.04**	0.02			0.00	0.02
(13) Other			−0.01	0.01			−0.01	0.01
Study day	−0.00	0.00	−0.00	0.00	**−0.01**	0.00	−**0.01**	0.00
Weekend (1 = yes)			0.02	0.02			−0.02	0.02
Sex (1 = men)			**0.26**	0.13			−**0.20**	0.09
Years of education			−**0.04**	0.02			0.00	0.01
Marital status (1 = married)			0.12	0.12			−0.06	0.09
Age			−0.01	0.01			0.01	0.01
Self-rated health			−**0.32**	0.07			**0.18**	0.05
Random effects								
Intercept VAR	0.47		0.39		0.24		0.21	
Slope (diversity WP) VAR	0.00		0.00		0.00		0.00	
Intercept-slope corr	0.00		−0.09		−0.01		0.00	
Residual VAR	0.16		0.16		0.12		0.12	
Marginal *R*^2^/conditional *R*^2^	0.01/0.75		0.17/0.76		0.01/0.67		0.12/0.68	

*Notes: b* = mean estimate; diversity BP = activity diversity between persons; diversity WP = activity diversity within persons; *SE* = standard error; VAR = variance; (1) housework = housework/gardening/administrative tasks (at home)/shopping; (2) cook/eat = cooking/preparing food/eating; (3) TV/music = watching TV/listening to music; (4) education/intellectual = education/intellectual stimulation (e.g., reading, puzzles, further education); (5) cultural/religious = cultural/religious activity (e.g., museum, cinema, church); (6) hobbies = hobby (e.g., crafts, handicrafts, making music); (7) social = social interactions (e.g., conversations, visits); (8) sports = sporting activity; (9) walk = going for a walk; (10) doctor/care = visiting the doctor/personal care (incl. e.g., hairdresser); (11) work/volunteer = work/voluntary work; (12) rest = doing nothing/resting; (13) other = other activity; VAR = variance; corr = correlation; Marginal *R*^2^ = the proportion of total outcome variance explained by fixed factors; conditional *R*^2^ = the proportion of total outcome variance explained by both fixed and random factors. Boldface estimates are statistically significant (*p* < .05).

**Figure 1.**
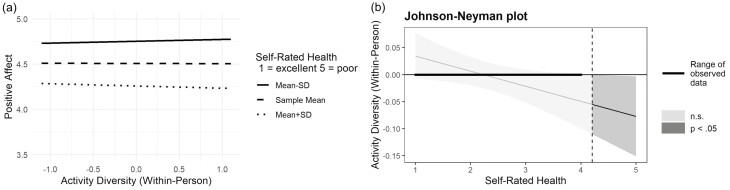
Plots of moderating effect of self-rated health on within-person association between daily activity diversity and daily positive affect. n.s. = not significant; SD = standard deviation.

As shown in Table 3, we found a significant moderating effect of self-rated health. Participants with lower self-rated health showed a negative within-person association between daily activity diversity and daily positive affect (Model 3, *b* = −0.03, *SE* = 0.01, *p* = .040). Yet, analysis with the Johnson-Neyman technique showed that the regions of significance of the moderating effect were either below −1.08 or above 4.45, which were outside the range of scores for the sample (Figure 1, panel B). More specifically, our participants rated from 1 (= excellent) to 4 (= fair) on the self-rated health scale (range = 1 to 5 [= poor]). In other words, the negative within-person association between daily activity diversity and daily positive affect could be observed in participants who rated their own health as “poor.” Additionally, this finding is seen in Figure 1, panel A, in which the lines representing within-person association between daily activity diversity and daily positive affect were almost horizontal and parallel across participants of different self-rated health levels.

**Table 3.** Associations Between Daily Activity Diversity and Daily Well-Being by Moderators

Parameters	Model 1 Positive affect		Model 2 Negative affect		Model 3 Positive affect		Model 4 Negative affect	
	*b*	*SE*	*b*	*SE*	*b*	*SE*	*b*	*SE*
Fixed effects								
Intercept	**4.30**	0.10	**0.79**	0.07	**4.32**	0.09	**0.79**	0.07
Diversity WP	−0.00	0.01	0.00	0.01	−0.00	0.01	0.00	0.01
Diversity BP	0.11	0.10	0.04	0.07	0.07	0.10	0.06	0.07
Age	−0.01	0.01	0.01	0.01				
Diversity WP × age	0.00	0.00	−0.00	0.00				
Diversity BP × age	0.01	0.02	−0.01	0.01				
Health					−**0.31**	0.07	**0.18**	0.05
Diversity WP × health					−**0.03**	0.01	0.01	0.01
Diversity BP × health					0.10	0.11	−0.08	0.08
(1) Housework	0.01	0.01	−**0.04**	0.01	0.01	0.01	−**0.04**	0.01
(2) Cook/eat	0.01	0.01	−**0.04**	0.01	0.01	0.01	−**0.04**	0.01
(3) TV/music	0.00	0.02	−**0.04**	0.01	0.00	0.02	−**0.04**	0.01
(4) Education/intellectual	0.01	0.01	−**0.05**	0.01	0.01	0.01	−**0.05**	0.01
(5) Cultural/religious	0.03	0.04	−0.05	0.04	0.03	0.04	−0.05	0.04
(6) Hobbies	0.02	0.02	−0.03	0.02	0.02	0.02	−0.03	0.02
(7) Social	**0.03**	0.01	−**0.03**	0.01	**0.03**	0.01	−**0.03**	0.01
(8) Sports	**0.08**	0.02	−**0.05**	0.02	**0.08**	0.02	−**0.05**	0.02
(9) Walk	**0.07**	0.03	−**0.07**	0.02	**0.07**	0.03	−**0.07**	0.02
(10) Doctor/care	−0.05	0.03	0.02	0.03	−0.05	0.03	0.02	0.03
(11) Work/volunteer	−0.02	0.02	−**0.04**	0.02	−0.02	0.02	−**0.04**	0.02
(12) Rest	−**0.04**	0.02	0.00	0.02	−**0.04**	0.02	0.00	0.02
(13) Other	−0.01	0.01	−0.01	0.01	−0.01	0.01	−0.01	0.01
Study day	−0.00	0.00	−**0.01**	0.00	−0.00	0.00	−**0.01**	0.00
Weekend (1 = yes)	0.02	0.02	−0.02	0.02	0.02	0.02	−0.02	0.02
Sex (1 = men)	**0.27**	0.14	−**0.21**	0.10	**0.24**	0.12	−0.17	0.09
Years of education	−0.03	0.02	0.00	0.01	−**0.04**	0.02	−0.00	0.01
Marital status (1 = married)	0.17	0.13	−0.07	0.09	0.15	0.12	−0.10	0.09
Random effects (VAR)								
Intercept VAR	0.46		0.23		0.39		0.21	
Slope (diversity WP) VAR	0.00		0.00		0.00		0.00	
Intercept-slope corr	0.05		0.06		−0.10		−0.01	
Residual VAR	0.16		0.12		0.16		0.12	
Marginal *R*^2^/conditional *R*^2^	0.08/0.76		0.07/0.68		0.17/0.76		0.12/0.68	

*Notes: b* = mean estimate; diversity BP = activity diversity between persons; diversity WP = activity diversity within persons; health = self-reported health; *SE* = standard error; VAR = variance; (1) housework = housework/gardening/administrative tasks (at home)/shopping; (2) cook/eat = cooking/preparing food/eating; (3) TV/music = watching TV/listening to music; (4) education/intellectual = education/intellectual stimulation (e.g., reading, puzzles, and further education); (5) cultural/religious = cultural/religious activity (e.g., museum, cinema, and church); (6) hobbies = hobby (e.g., crafts, handicrafts, and making music); (7) social = social interactions (e.g., conversations and visits); (8) sports = sporting activity; (9) walk = going for a walk; (10) doctor/care = visiting the doctor/personal care (incl. e.g., hairdresser); (11) work/volunteer = work/voluntary work; (12) rest = doing nothing/resting; (13) other = other activity; VAR = variance; corr = correlation; marginal *R*^2^ = the proportion of total outcome variance explained by fixed factors; conditional *R*^2^ = the proportion of total outcome variance explained by both fixed and random factors. Boldface estimates are statistically significant (*p* < .05).

## Discussion and Conclusion

With 2-week smartphone-based ambulatory assessment data from community-dwelling older adults, we examined associations between activity diversity and well-being of older adults. Different from our expectations and previous results in the literature, we did not find any association of daily activity diversity with daily positive or negative affect at the between- or within-person levels. Moderation analysis showed that participants with lower self-rated health experienced lower positive affect on a day when they had more diverse activities than usual. However, analysis with the Johnson-Neyman technique showed that the region of significance of the moderating effect was only significant in participants who rated their health as “poor” and was outside the observed data.

**Figure 2.**
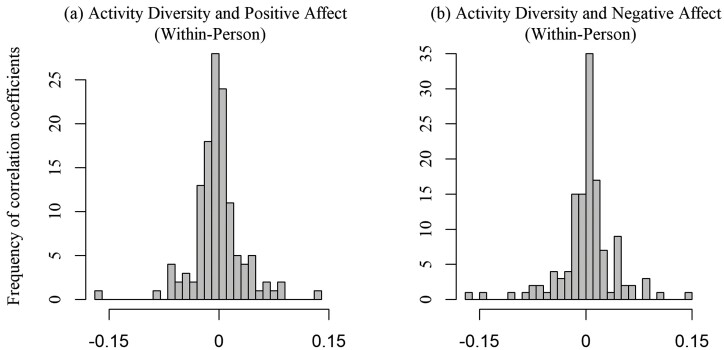
Histogram of person-specific coefficients of within-person associations between daily activity diversity and daily positive and negative affect. The histograms represent the coefficients for each participant relating their daily positive and negative affect to daily activity diversity at the within-person level. In panel (**A**), the correlation coefficients ranged from −0.17 to 0.13. In panel (**B**), the correlation coefficients ranged from −0.16 to 0.15.

Referring to prior research that showed a positive between-person association of activity diversity with well-being (Lee, Koffer, et al., 2018), we expected a positive between-person association in our data. However, we did not find any significant relations indicting that interindividual differences in activity diversity were linked with differences in emotional well-being. Further, we examined the association at the within-person level to understand whether beyond person-level differences there were meaningful intraindividual covariations in daily life, and did not find any significant relations. To understand our pattern of not statistically significant results, we plotted Figure 2 to examine the degree of heterogeneity of within-person relations as indicated by the histograms of coefficients for each individual participant relating their daily positive and negative affect to daily activity diversity (based on Table 2, Models 2 and 4). This plot indicates divergent patterns in the within-person associations across participants. That is, activity diversity relates to higher well-being in the daily lives of some participants, but to lower well-being in others. Considering such divergent patterns explained, the finding of a nonsignificant overall sample average estimate of the within-person associations is plausible.

Next, we examined whether age and self-rated health could explain some of the observed heterogeneity by introducing them as between-person moderators of the within-person associations between activity diversity and well-being. Referring to the selective optimization with compensation model (Baltes & Carstensen, 1996) and the socioemotional selectivity theory (Carstensen, 1995), we expected that older age and lower self-rated health may motivate more careful selection in daily activities and, thus, would lead to lower well-being in light of more diverse activities at both the within- and between-person levels. Results showed that self-rated health moderated the association of daily activity diversity and daily positive affect at the within-person level, but the moderating effect was not significant within the range of observed data. That is, older adults’ “poor” health ratings may express a lower availability of personal resources and lower energy levels, which prompt them to be selective in their pursuit of daily activities, leading to less pleasant experience on a day with more diverse activities. This finding is in line with recent research, which proposed that energy depletion and its recovery should be taken into account when understanding the full complexity of activity engagement in older age (Cardini & Freund, 2019; Luo, Pauly, et al., 2022). However, the scores of self-rated health in our study were better than “poor” and did not fall within the region of significance. Thus, we could not generalize the moderating effect to our study sample.

Markedly, we found a negative within-person association between daily activity diversity and daily negative affect at first, but the effects became nonsignificant once we controlled for the effects of the frequency of several activities. An intervention study to boost happiness showed that diverse positive activities were associated with higher well-being, but the association reversed when total activity frequency was taken into account (Okabe-Miyamoto et al., 2023). This finding speaks to the importance of considering activity *frequency* in the examination of activity *diversity*. We also noted that the prior study indicating a between-person association of activity diversity with well-being controlled for the total duration of activity participation (Lee, Koffer, et al., 2018). Said prior study calculated the total duration of all activities as one covariate, whereas our study calculated the frequency of different activities separately. Activity duration across all activities may have given more weight to the activities that occupied more time, but not necessarily reflected the respective effects of activities that were important for older adults’ well-being.

Additionally, our findings showed that higher frequency of social interactions (item 7), sports activities (item 8), and walking (item 9) were associated with higher positive affect (Table 2, Model 2), whereas higher frequency of the aforementioned activities and, additionally, housework/gardening/shopping (item 1), cooking/eating (item 2), watching television/listening to music (item 3), and education/mental stimulation (item 4) were associated with lower negative affect (Table 2, Model 4). The findings on sociocognitive (education/mental stimulation [item 4], social interactions [item 7]), and physical activities (sports activities [item 8], walking [item 9]) are in line with existing research on older adults’ activity engagement and well-being (Adams et al., 2011; Netz et al., 2005). These activities were also rated by midaged adults as meaningful activities, and their engagement was associated with greater positive affect (Hooker et al., 2020).

The findings on housework/gardening/shopping (item 1), cooking/eating (item 2), and watching television/listening to music (item 3) were inconsistent with prior research. Some research showed that television watching was related to lower positive affect and higher loneliness (Fingerman et al., 2022; Goodwin et al., 2005), although a paper showed that television watching was related to fewer stressors in life (Charles, Mogle, et al., 2021). It has been reported that household activities, including preparing meals and grocery shopping, were related to lower happiness (Oerlemans et al., 2011). We assumed that associations between activity frequency and well-being might also depend on social contexts, which we did not specifically consider in this article. For example, older adults have been found to spend more time watching television when they are alone than when they are with other people (Fingerman et al., 2022). This could be similar to other activities (e.g., cooking, shopping, and television watching) in such that the experience of an activity is altered by the presence of others.

We acknowledge several limitations in this study. First, although this study tried to capture activities that commonly occur in daily life, the choices offered for selection were still limited as a function of balancing our quest for information with participant burden in seven repeated assessments per day over 2 weeks. We also did not request participants to report their exact activity for the item of “other activities” (8% of the total observations). Future studies could consider adding more activities, such as grandparental childcare. Moreover, there could be variations across participants in how a particular activity was categorized. For example, visiting a cinema with a friend may be a social activity for some participants, but a cultural activity for others. There might also be other elements of activities that are important for older adults’ well-being and warrant future investigation, such as novelty (Chu et al., 2024), meaningfulness (Hooker et al., 2020), enjoyment, and importance (Jarosz, 2022).

Second, some of the activities had relatively lower frequency observed, such as doctor’s visits. This may influence the results of our findings on activity frequency. Relatedly, our design with the assessment at seven times per day may miss activities that occurred outside of these timepoints. Future research could use alternative (e.g., event-based or sensing) methods to continuously and possibly unobtrusively capture everyday activities over longer times (Hülür et al., 2023; Ng et al., 2021). Third, associations between activities and well-being could have been dependent on activity contexts, such as cooking alone versus eating with others. It is beyond the scope of this article to incorporate the contexts of activity (Charles, Röcke, et al., 2021), but future research could address the effects of contexts on the associations between activity diversity/frequency and well-being. Finally, we did not observe the hypothesized association between activity diversity and well-being and did not find the expected moderating effects of age and self-rated health at the between-person level. This might be due to the sample characteristics of the relatively active and high-functioning community-dwelling Swiss older adult participants. Future research would profit from more heterogeneous samples in terms of health status and social background.

To conclude, this study examined the associations between activity diversity and well-being on a day-to-day basis. Our findings suggest that rather than a uniform associative picture, there were quite divergent patterns in associations between daily activity diversity and daily well-being across individuals and, thus, not everybody experienced increased or decreased well-being on a day with diverse activities. Potentially, older adults with “poor” self-rated health may experience less positive affect on days with more diverse activities than usual, suggesting the experience of a suboptimal mapping between available resources and situational challenges. Yet, this proposition should be examined with further evidence. Taken together, our findings shed light on the discussion of how to optimize and regulate time spent in older age to maintain well-being (Horgas et al., 1998; Luo, Pauly, et al., 2022; Möwisch et al., 2022).

## Supplementary Material

gbae025_suppl_Supplementary_Material

## Data Availability

This study was not preregistered. The data that support the findings of this study are not publicly available because the data belong to an ongoing longitudinal study. The data and the detailed analyses are available upon reasonable request from the data sharing committee via Dr. C. Röcke (christina.roecke@uzh.ch).

## Author Note

1. As an additional analysis, we examined the data of all 150 participants and excluded the item of work in the calculation of activity diversity. Results were the same.

## References

[CIT0001] Adams, K. B., Leibbrandt, S., & Moon, H. (2011). A critical review of the literature on social and leisure activity and wellbeing in later life. Ageing & Society, 31(4), 683–712. 10.1017/s0144686x10001091

[CIT0002] Baltes, M. M., & Carstensen, L. L. (1996). The process of successful ageing. Ageing & Society, 16(4), 397–422. 10.1017/s0144686x00003603

[CIT0003] Barton, K., & Barton, M. K. (2015). Package “mumin.”.Version, 1(18), 439. https://cran.hafro.is/web/packages/MuMIn/MuMIn.pdf

[CIT0004] Bates, D., Sarkar, D., Bates, M. D., & Matrix, L. (2007). The lme4 package. R Package Version, 2(1), 74. https://cran.r-project.org/web/packages/lme4/lme4.pdf

[CIT0006] Bolger, N., & Laurenceau, J.-P. (2013). Intensive longitudinal methods: An introduction to diary and experience sampling research. Guilford Press.

[CIT0007] Brown, C. J., Jeon, S., Ng, Y. T., Lee, S., Fingerman, K. L., & Charles, S. T. (2023). Switching it up: Activity diversity and cognitive functioning in later life. Psychology and Aging, 38(6), 483–493. 10.1037/pag0000770 37535516 PMC10528947

[CIT0008] Buchman, A. S., Wilson, R. S., Yu, L., James, B. D., Boyle, P. A., & Bennett, D. A. (2014). Total daily activity declines more rapidly with increasing age in older adults. Archives of Gerontology and Geriatrics, 58(1), 74–79. 10.1016/j.archger.2013.08.001 24007938 PMC3889486

[CIT0009] Cardini, B. B., & Freund, A. M. (2019). When the fun is over. European Psychologist, 24(4), 322–336. 10.1027/1016-9040/a000361

[CIT0010] Carstensen, L. L. (1995). Evidence for a life-span theory of socioemotional selectivity. Current Directions in Psychological Science, 4(5), 151–156. 10.1111/1467-8721.ep11512261 PMC8340497 34366582

[CIT0011] Charles, S., Röcke, C., Zadeh, R. S., Martin, M., Boker, S., & Scholz, U. (2021). Leveraging daily social experiences to motivate healthy aging. Journals of Gerontology, Series B: Psychological Sciences and Social Sciences, 76(Suppl_2), S157–S166. 10.1093/geronb/gbab028 33861858

[CIT0012] Charles, S. T., Mogle, J., Chai, H. W., & Almeida, D. M. (2021). The mixed benefits of a stressor-free life. Emotion, 21(5), 962–971. 10.1037/emo0000958 33630624 PMC8384975

[CIT0059] Chu, L., Shavit, Y. Z., Ram, N., & Carstensen, L. L. (2024). Age-related emotional advantages in encountering novel situation in daily life. *Psychology and Aging*, 39(2), 113–125. 10.1037/pag0000798 38436654

[CIT0013] Fingerman, K. L., Huo, M., Charles, S. T., & Umberson, D. J. (2020). Variety is the spice of late life: Social integration and daily activity. Journals of Gerontology, Series B: Psychological Sciences and Social Sciences, 75(2), 377–388. 10.1093/geronb/gbz007 30783671 PMC7179804

[CIT0014] Fingerman, K. L., Kim, Y. K., Ng, Y. T., Zhang, S., Huo, M., & Birditt, K. S. (2022). Television viewing, physical activity, and loneliness in late life. Gerontologist, 62(7), 1006–1017. 10.1093/geront/gnab120 34379115 PMC9372884

[CIT0015] Finkel, D., Andel, R., & Pedersen, N. L. (2018). Gender differences in longitudinal trajectories of change in physical, social, and cognitive/sedentary leisure activities. Journals of Gerontology, Series B: Psychological Sciences and Social Sciences, 73(8), 1491–1500. 10.1093/geronb/gbw116 27624718 PMC6890525

[CIT0016] Fisher, K., Gershuny, J., & Gauthier, A. (2012). Multinational time use study: User’s guide and documentation. Centre for Time Use Research, University of Oxford.

[CIT0017] Freund, A. M. (2020). The bucket list effect: Why leisure goals are often deferred until retirement. American Psychologist, 75(4), 499–510. 10.1037/amp0000617 32378945

[CIT0018] Goodwin, P. E., Intrieri, R. C., & Papini, D. R. (2005). Older adults’ affect while watching television. Activities, Adaptation & Aging, 29(2), 55–72. 10.1300/j016v29n02_04

[CIT0019] Grühn, D., Kotter-Grühn, D., & Röcke, C. (2010). Discrete affects across the adult lifespan: Evidence for multidimensionality and multidirectionality of affective experiences in young, middle-aged and older adults. Journal of Research in Personality, 44(4), 492–500. 10.1016/j.jrp.2010.06.003

[CIT0020] Guralnik, J. M., Simonsick, E. M., Ferrucci, L., Glynn, R. J., Berkman, L. F., Blazer, D. G., Scherr, P. A., & Wallace, R. B. (1994). A short physical performance battery assessing lower extremity function: Association with self-reported disability and prediction of mortality and nursing home admission. Journal of Gerontology, 49(2), M85–94. 10.1093/geronj/49.2.m85 8126356

[CIT0021] Havighurst, R. J. (1963). Successful aging. In R. H. Williams, C. Tibbitts, & W. Donohue (Eds.), Processes of aging: Social and psychological perspectives (Vol. 1, pp. 299–320). Transaction Publishers.

[CIT0022] Hooker, S. A., Masters, K. S., Vagnini, K. M., & Rush, C. L. (2020). Engaging in personally meaningful activities is associated with meaning salience and psychological well-being. Journal of Positive Psychology, 15(6), 821–831. 10.1080/17439760.2019.1651895

[CIT0023] Horgas, A. L., Wilms, H. -U., & Baltes, M. M. (1998). Daily life in very old age: Everyday activities as expression of successful living. Gerontologist, 38(5), 556–568. 10.1093/geront/38.5.556 9803644

[CIT0024] Hultsch, D. F., Hertzog, C., Small, B. J., & Dixon, R. A. (1999). Use it or lose it: Engaged lifestyle as a buffer of cognitive decline in aging? Psychology and Aging, 14(2), 245–63. 10.1037//0882-7974.14.2.245 10403712

[CIT0025] Hülür, G., Luo, M., Macdonald, B., & Grünjes, C. E. (2023). The perceived quality of social interactions differs by modality and purpose: An event-contingent experience sampling study with older adults. Journal of Social and Personal Relationships, 41(4), 794–821. 10.1177/02654075231215269

[CIT0026] Janke, M., Davey, A., & Kleiber, D. (2006). Modeling change in older adults’ leisure activities. Leisure Sciences, 28(3), 285–303. 10.1080/01490400600598145

[CIT0027] Jansen, D. A., Sauve, J. L., & Aubart, S. M. (2023). Importance of variety to the lives and wellbeing of elders. Activities, Adaptation & Aging, 47, 461–481. 10.1080/01924788.2023.2174732

[CIT0028] Jarosz, E. (2022). What makes life enjoyable at an older age? Experiential wellbeing, daily activities, and satisfaction with life in general. Aging & Mental Health, 26(6), 1242–1252. 10.1080/13607863.2021.1916879 33908290

[CIT0029] Joseph Sirgy, M., & Wu, J. (2009). The pleasant life, the engaged life, and the meaningful life: What about the balanced life? Journal of Happiness Studies, 10, 183–196. 10.1007/s10902-007-9074-1

[CIT0030] Kim, Y. K., & Fingerman, K. L. (2022). Daily social media use, social ties, and emotional well-being in later life. Journal of Social and Personal Relationships, 39(6), 1794–1813. 10.1177/02654075211067254 37727534 PMC10508904

[CIT0031] Kuznetsova, A., Brockhoff, P. B., & Christensen, R. H. (2017). lmerTest package: Tests in linear mixed effects models. Journal of Statistical Software, 82(1), 1–26. 10.18637/jss.v082.i13

[CIT0033] Lee, H. -Y., Yu, C. -P., Wu, C. -D., & Pan, W. -C. (2018). The effect of leisure activity diversity and exercise time on the prevention of depression in the middle-aged and elderly residents of Taiwan. International Journal of Environmental Research and Public Health, 15(4), 654. 10.3390/ijerph15040654 29614768 PMC5923696

[CIT0034] Lee, S., Koffer, R. E., Sprague, B. N., Charles, S. T., Ram, N., & Almeida, D. M. (2018). Activity diversity and its associations with psychological well-being across adulthood. Journals of Gerontology, Series B: Psychological Sciences and Social Sciences, 73(6), 985–995. 10.1093/geronb/gbw118 27621306 PMC6454790

[CIT0035] Lee, S., Ng, Y. T., Charles, S. T., Almeida, D. M., & Fingerman, K. L. (2022). Who has active lifestyles? Sociodemographic and personality correlates of activity diversity in two samples of adults. Journals of Gerontology, Series B: Psychological Sciences and Social Sciences, 78, 659–669. 10.1093/geronb/gbac192 PMC10066737 36512323

[CIT0036] Lee, S., Urban-Wojcik, E. J., Charles, S. T., & Almeida, D. M. (2022). Rich and balanced experiences of daily emotions are associated with activity diversity across adulthood. Journals of Gerontology, Series B: Psychological Sciences and Social Sciences, 77(4), 710–720. 10.1093/geronb/gbab144 34343286 PMC8974333

[CIT0037] Lindner, S., Stieger, M., Rüegger, D., Kowatsch, T., Flückiger, C., Mehl, M. R., & Allemand, M. (2023). How is variety in daily life related to the expression of personality states? An ambulatory assessment study. European Journal of Personality, 38, 172–188. 10.1177/08902070221149593 PMC13029511 41908318

[CIT0038] Long, J. A., & Long, M. J. A. (2019). Package “interactions.” *See* https://pbil.univ-lyon1.fr/CRAN/web/packages/interactions/interactions.pdf

[CIT0039] Luo, M., Macdonald, B., & Hülür, G. (2022). Not “The More The Merrier”: Diminishing returns to daily face-to-face social interaction frequency for well-being in older age. Journals of Gerontology, Series B: Psychological Sciences and Social Sciences, 77(8), 1431–1441. 10.1093/geronb/gbac010 35077534

[CIT0040] Luo, M., Moulder, R. G., Breitfelder, L. K., & Röcke, C. (2023). Daily activity diversity and daily working memory in community-dwelling older adults. Neuropsychology, 37, 181–193. 10.1037/neu0000878 36689393

[CIT0041] Luo, M., Pauly, T., Röcke, C., & Hülür, G. (2022). Alternating time spent on social interactions and solitude in healthy older adults. British Journal of Psychology (London, England: 1953), 113(4), 987–1008. 10.1111/bjop.12586 35957493 PMC9804578

[CIT0042] Möwisch, D., Brose, A., & Schmiedek, F. (2022). Active time use and well-being in older adulthood: Results from a day reconstruction method study. Work, Aging and Retirement, 9, 7–18. 10.1093/workar/waab030

[CIT0043] Netz, Y., Wu, M. -J., Becker, B. J., & Tenenbaum, G. (2005). Physical activity and psychological well-being in advanced age: A meta-analysis of intervention studies. Psychology and Aging, 20(2), 272–84. 10.1037/0882-7974.20.2.272 16029091

[CIT0044] Ng, Y. T., Huo, M., Han, S. H., Birditt, K., & Fingerman, K. (2021). Older adult’s marital status, conversation frequency, and well-being in everyday life. Journals of Gerontology, Series B: Psychological Sciences and Social Sciences, 77(3), 499–512. 10.1093/geronb/gbab112 PMC8893135 34159387

[CIT0045] Oerlemans, W. G., Bakker, A. B., & Veenhoven, R. (2011). Finding the key to happy aging: A day reconstruction study of happiness. Journals of Gerontology. Series B, Psychological Sciences and Social Sciences, 66(6), 665–674. 10.1093/geronb/gbr040 21724970

[CIT0046] Okabe-Miyamoto, K., Margolis, S., & Lyubomirsky, S. (2023). Is variety the spice of happiness? More variety is associated with lower efficacy of positive activity interventions in a sample of over 200,000 happiness seekers. Journal of Positive Psychology, 18(3), 327–338. 10.1080/17439760.2021.2006760

[CIT0047] Preacher, K. J., Curran, P. J., & Bauer, D. J. (2006). Computational tools for probing interactions in multiple linear regression, multilevel modeling, and latent curve analysis. Journal of Educational and Behavioral Statistics, 31(4), 437–448. 10.3102/10769986031004437

[CIT0048] R Core Team. (2013). *R: A language and environment for statistical computing*.

[CIT0049] Röcke, C., Luo, M., Bereuter, P., Katana, M., Fillekes, M., Gehriger, V., Alexandros, S., Martin, M., & Weibel, R. (2023). Charting everyday activities in later life: Study protocol of the mobility, activity, and social interactions study (MOASIS). Frontiers in Psychology, 13, 1–20 10.3389/fpsyg.2022.1011177 PMC9903074 36760916

[CIT0050] Ruissen, G. R., Beauchamp, M. R., Puterman, E., Zumbo, B. D., Rhodes, R. E., Hives, B. A., Sharpe, B. M., Vega, J., Low, C. A., & Wright, A. G. (2022). Continuous-time modeling of the bidirectional relationship between incidental affect and physical activity. Annals of Behavioral Medicine, 56(12), 1284–1299. 10.1093/abm/kaac024 35802004 PMC9672348

[CIT0051] Sardina, A. L., Mahlobo, C. T., Gamaldo, A. A., Allaire, J. C., & Whitfield, K. E. (2022). Exploring the association between affect and leisure activity engagement in Black adults. Journals of Gerontology, Series B: Psychological Sciences and Social Sciences, 77(12), 2157–2169. 10.1093/geronb/gbac084 35772778 PMC9923799

[CIT0052] Shannon, C. E. (1948). A mathematical theory of communication. Bell System Technical Journal, 27(3), 623–656. 10.1002/j.1538-7305.1948.tb00917.x

[CIT0053] Takahashi, J., Kawai, H., Ejiri, M., Fujiwara, Y., Hirano, H., Sasai, H., Ihara, K., Ishii, K., Oka, K., & Obuchi, S. (2023). Activity diversity is associated with the prevention of frailty in community-dwelling older adults: The Otassha Study. Frontiers in Public Health, 11, 1–7. 10.3389/fpubh.2023.1113255 PMC10078622 37033071

[CIT0054] Taquet, M., Quoidbach, J., De Montjoye, Y. -A., Desseilles, M., & Gross, J. J. (2016). Hedonism and the choice of everyday activities. Proceedings of the National Academy of Sciences of the United States of America, 113(35), 9769–9773. 10.1073/pnas.1519998113 27528666 PMC5024602

[CIT0055] Ware Jr, J. E., Kosinski, M., & Keller, S. D. (1996). A 12-Item Short-Form Health Survey: Construction of scales and preliminary tests of reliability and validity. Medical Care, 34, 220–233. 10.1097/00005650-199603000-00003. 8628042

[CIT0056] Watson, D., Clark, L. A., & Tellegen, A. (1988). Development and validation of brief measures of positive and negative affect: The PANAS scales. Journal of Personality and Social Psychology, 54(6), 1063–70. 10.1037//0022-3514.54.6.1063 3397865

[CIT0057] Wilhelm, P., & Schoebi, D. (2007). Assessing mood in daily life. European Journal of Psychological Assessment, 23(4), 258–267. 10.1027/1015-5759.23.4.258

[CIT0058] World Health Organization. (2015). World report on ageing and health. Author.

