# Supplementary material for: Activity Diversity and Well-Being in Daily Life: Evidence for Heterogeneity Between Older Adults
Source: J Gerontol B Psychol Sci Soc Sci. 2024 Mar 30;79(6):gbae025. doi: 10.1093/geronb/gbae025 (PMC11075729; doi:10.1093/geronb/gbae025)
Supplement: gbae025_suppl_Supplementary_Material [file gbae025_suppl_supplementary_material.docx]

**Supplementary Materials**

Table S1. *Bivariate Between-Person Associations of the Focal Variables.*

| Variable | 1 | 2 | 3 | 4 | 5 | 6 | 7 | 8 | 9 | 10 | 11 | 12 | 13 | 14 | 15 | 16 | 17 | 18 | 19 | 20 | 21 | 22 |
| --- | --- | --- | --- | --- | --- | --- | --- | --- | --- | --- | --- | --- | --- | --- | --- | --- | --- | --- | --- | --- | --- | --- |
| 1. Positive affect |  |  |  |  |  |  |  |  |  |  |  |  |  |  |  |  |  |  |  |  |  |  |
| 2. Negative affect | **-.68** |  |  |  |  |  |  |  |  |  |  |  |  |  |  |  |  |  |  |  |  |  |
| 3. Activity diversity | .16 | -.08 |  |  |  |  |  |  |  |  |  |  |  |  |  |  |  |  |  |  |  |  |
| 4. (1) Housework | .14 | -.10 | **.27** |  |  |  |  |  |  |  |  |  |  |  |  |  |  |  |  |  |  |  |
| 5. (2) Cook/Eat | **.23** | **-.18** | **.43** | **.21** |  |  |  |  |  |  |  |  |  |  |  |  |  |  |  |  |  |  |
| 6. (3) TV/Music | .17 | -.09 | **.33** | .03 | .11 |  |  |  |  |  |  |  |  |  |  |  |  |  |  |  |  |  |
| 7. (4) Education/Intellectual | -.03 | .04 | **.30** | -.13 | -.07 | .03 |  |  |  |  |  |  |  |  |  |  |  |  |  |  |  |  |
| 8. (5) Cultural/Religious | -.00 | .01 | **.25** | .07 | -.05 | -.10 | .08 |  |  |  |  |  |  |  |  |  |  |  |  |  |  |  |
| 9. (6) Hobbies | .07 | -.14 | .06 | -.03 | .04 | -.17 | -.03 | -.02 |  |  |  |  |  |  |  |  |  |  |  |  |  |  |
| 10. (7) Social | -.01 | .08 | **.32** | -.02 | .12 | -.11 | .11 | **.22** | .08 |  |  |  |  |  |  |  |  |  |  |  |  |  |
| 11. (8) Sports | .05 | .04 | **.24** | -.08 | .10 | .06 | .10 | -.12 | -.01 | -.06 |  |  |  |  |  |  |  |  |  |  |  |  |
| 12. (9) Walk | -.02 | .02 | **.29** | -.01 | .10 | -.10 | -.10 | **.30** | -.10 | **.24** | -.04 |  |  |  |  |  |  |  |  |  |  |  |
| 13. (10) Doctor/Care | -.15 | .11 | **.34** | .05 | -.03 | .08 | .10 | -.04 | -.03 | .05 | .08 | .05 |  |  |  |  |  |  |  |  |  |  |
| 14. (11) Work/Volunteer | .04 | -.15 | .15 | -.11 | .08 | -.09 | -.11 | .02 | -.00 | .02 | -.13 | .06 | .08 |  |  |  |  |  |  |  |  |  |
| 15. (12) Rest | .16 | -.03 | **.27** | -.03 | -.05 | .04 | .07 | .05 | .13 | -.08 | -.01 | -.08 | -.01 | .10 |  |  |  |  |  |  |  |  |
| 16. (13) Other | -.01 | -.05 | **.23** | -.01 | -.07 | .08 | -.09 | .08 | -.08 | -.08 | -.11 | .12 | .02 | -.02 | .03 |  |  |  |  |  |  |  |
| 17. Study Day | .05 | -.17 | **.44** | **.22** | **.25** | .10 | **.17** | .10 | -.03 | .15 | .05 | .10 | **.18** | .02 | .02 | .15 |  |  |  |  |  |  |
| 18. Weekday | .00 | .13 | **-.42** | **-.22** | **-.25** | -.17 | **-.18** | -.09 | -.05 | -.11 | -.06 | -.15 | -.15 | -.00 | -.03 | -.10 | **-.81** |  |  |  |  |  |
| 19. Sex | **.20** | **-.21** | .08 | **-.19** | .07 | .07 | .06 | .01 | .13 | -.12 | **.18** | -.08 | -.04 | **.34** | .11 | -.03 | -.02 | -.08 |  |  |  |  |
| 20. Years of education | -.10 | -.08 | -.04 | .05 | .11 | -.14 | -.06 | .10 | .02 | -.02 | -.11 | -.02 | .09 | .15 | **-.26** | **.18** | .04 | -.03 | **.23** |  |  |  |
| 21. Marital status | **.19** | **-.18** | .02 | .13 | **.28** | .06 | -.01 | -.10 | .07 | -.14 | .11 | -.07 | -.05 | .06 | -.12 | -.15 | .01 | -.07 | **.34** | .11 |  |  |
| 22. Self-rated health | **-.37** | **.31** | -.00 | -.03 | -.15 | .03 | -.12 | **.19** | -.15 | .06 | .02 | **.19** | .08 | -.11 | -.13 | .07 | -.05 | .03 | -.04 | -.06 | -.09 |  |
| 23. Age | -.07 | .15 | -.14 | -.08 | -.13 | .13 | .07 | -.08 | .02 | -.17 | -.10 | -.16 | -.05 | -.16 | .12 | -.05 | -.12 | .03 | .14 | -.11 | -.14 | .08 |

*Note.* (1) Housework = housework/gardening/administrative tasks (at home) / shopping; (2) Cook / Eat = cooking / preparing food / eating; (3) TV / Music = watching TV / listening to music; (4) Education / Intellectual = education / intellectual stimulation (e.g. reading, puzzles, further education); (5) Cultural / Religious = cultural / religious activity (e.g. museum, cinema, church); (6) Hobbies = hobby (e.g. crafts, handicrafts, making music); (7) Social = social interactions (e.g. conversations, visits); (8) Sports = sporting activity; (9) Walk = going for a walk; (10) Doctor / Care = visiting the doctor / personal care (incl. e.g. hairdresser); (11) Work / Volunteer = work / voluntary work; (12) Rest = doing nothing / resting; (13) Other = other activity.

Boldface estimates are statistically significant (*p* < .05).
